# The Role of Protein Modifications of T-Bet in Cytokine Production and Differentiation of T Helper Cells

**DOI:** 10.1155/2014/589672

**Published:** 2014-05-13

**Authors:** Sera Oh, Eun Sook Hwang

**Affiliations:** College of Pharmacy, School of Pharmaceutical Sciences and Global Top 5 Research Program, Ewha Womans University, 52 Ewhayeodae-gil, Seodaemun-gu, C206 Science Building, Seoul 120-750, Republic of Korea

## Abstract

T-Bet (T-box protein expressed in T cells, also called as TBX21) was originally cloned as a key transcription factor involved in the commitment of T helper (Th) cells to the Th1 lineage. T-Bet directly activates IFN-*γ* gene transcription and enhances development of Th1 cells. T-Bet simultaneously modulates IL-2 and Th2 cytokines in an IFN-*γ*-independent manner, resulting in an attenuation of Th2 cell development. Numerous studies have demonstrated that T-bet plays multiple roles in many subtypes of immune cells, including B cell, dendritic cells, natural killer (NK) cells, NK T cells, and innate lymphoid cells. Therefore, T-bet is crucial for the development and coordination of both innate and adaptive immune responses. To fulfill these multiple roles, T-bet undergoes several posttranslational protein modifications, such as phosphorylation at tyrosine, serine, and threonine residues, and ubiquitination at lysine residues, which affect lineage commitment during Th cell differentiation. This review presents a current overview of the progress made in understanding the roles of various types of T-bet protein modifications in the regulation of cytokine production during Th cell differentiation.

## 1. Introduction

T-Bet (T-box protein expressed in T cells, also called TBX21) was firstly described in 2000 in a report examining the effects of T-bet on the differentiation of T helper 1 (Th1) cells [1]. For the past 15 years, many studies have examined the functions of T-bet and have revealed multiple roles for this protein during Th cell differentiation, with a focus on the molecular mechanisms involved, the novel functions of this transcription factor in innate immune cells, and T-bet-mediated modulation of inflammatory diseases [2–9]. It has been clarified that T-bet plays a critical role in the coordination of innate immunity and adaptive immunity and that it fulfills an important function in modulating chronic inflammatory diseases, including asthma and inflammatory bowel disease, by controlling a network of highly conserved genetic programs [10–12]. Thus, optimal regulation of T-bet expression and activity seems to be beneficial for preventing or treating chronic inflammation and autoimmune diseases.

Although attempts have been made at identifying the small molecules that control the expression and activity of T-bet that affect the T cell-mediated immune response, little progress has been made on this to date. Given the importance of T-bet in the immune regulation, elucidating the functional mechanisms underlying the multiple roles of T-bet would facilitate the development of novel therapeutic interventions for treating chronic inflammatory and autoimmune diseases. This review summarizes the current state of knowledge about the molecular mechanisms underlying the multiple roles played by T-bet in Th cell development.

## 2. Structure of T-Bet

**Figure 1:**
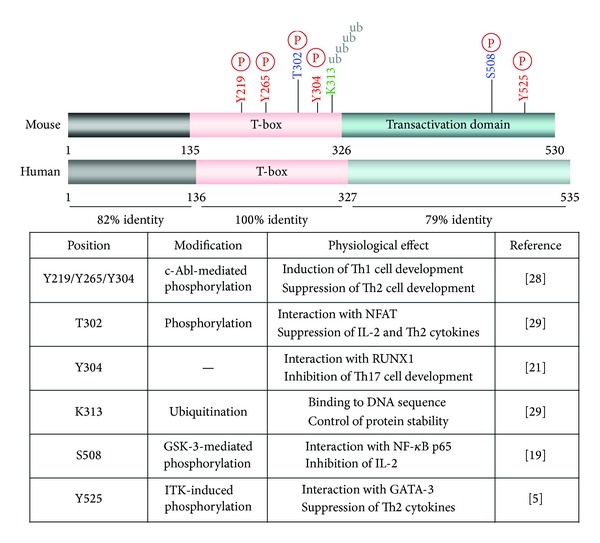
Structure and protein modification of T-bet. Mouse and human T-bet is 100% identical in the T-box domain. Several amino acid residues are conserved in mice and undergo posttranslational modifications, including phosphorylation at serine, threonine, and/or tyrosine residues, and ubiquitination at lysine residues.

The T-bet contains an amino-terminus, a T-box domain, and a carboxyl-terminus, which show 82%, 100%, and 79% homology, respectively, between mice (530 amino-acid residue protein) and humans (535 amino-acid residue protein) (Figure 1). The T-box domain, located between residues 135 and 326 in mouse T-bet, is highly conserved in 18 members of the T-box protein (TBX) family [13, 14]. Common features shared by T-box proteins include a capacity for DNA binding through the T-box domain and transcriptional regulatory activity, which plays a role in controlling the expression of developmental gene in all animal species.

The T-box domain is made up of about 180 amino-acid residues and is both sufficient and necessary for binding to the consensus DNA sequence TCACACCT [13–15]. Brachyury (T) was the first T-box protein to be identified and, in dimeric form, interacts with the major and the minor grooves of DNA through hydrophobic interactions and unusual main-chain carbonyl contact with a guanine as a dimer [16]. TBX1 also binds to the DNA sequence as a dimer, whereas TBX2 appears to bind to the same DNA sequence as a monomer [17]. Although TBX1 and TBX2 share 61% identity in the T-box domain, the structure of the DNA-T-box binding complex appears to be different, because of the low homology among the amino- and carboxyl-terminal regions. The T-box domain in T-bet shows 50% homology with the corresponding domain in brachyury (T), TBX1, and TBX2; however, the crystal structure of T-bet bound to the DNA sequence remains to be characterized.

## 3. Regulation of Th Cell Differentiation by T-Bet

### 3.1. Stimulation of Th1 Cell Differentiation by T-Bet

T-bet directly binds to the consensus DNA sequence within the* IFNG* promoter and activates its transcription. The T-bet-induced expression of* IFNG* derives Th precursor cells to differentiate into Th1 effector cells. While exogenous T-bet overexpression in naïve Th cells preferentially increases development of Th1 cells, T-bet deficiency leads to a failure to produce sufficient IFN-*γ* and therefore reduces generation of Th1 cells [1, 18]. T-Bet expression is substantially increased by stimulation of the T cell receptor (TCR) and is augmented by cotreatment with IFN-*γ* and IL-12. IFN-*γ* binds to its receptor and induces activation of signal transducer and activator of transcription (STAT) 1 and transcription of T-bet gene (*TBX21*). Subsequently, T-bet directly stimulates the transcription of* IFNG* as well as* IL12RB2*. Expression of IL-12 receptor (IL-12R) *β*2 on the cell surface further enhances IFN-*γ* production through IL-12 and the STAT4 signaling pathway, thereby resulting in preferential Th1 cell differentiation. Interestingly, enforced T-bet expression can also convert the differentiated Th2 cells into Th1 cells [1]. Therefore, T-bet is positioned at the crux of the regulatory pathways that induce IFN-*γ* in Th cells.

### 3.2. Attenuation of IL-2 Production by T-Bet

In addition to IFN-*γ* regulation, T-bet significantly suppresses IL-2 expression. This cytokine, an early T cell growth factor, is essential for activation, proliferation, and differentiation of Th cells and is abundantly produced upon TCR stimulation. Ectopically introduced T-bet significantly suppresses IL-2 production through inhibition of nuclear factor *κ*B (NF-*κ*B) p65 activity, under conditions of both Th1 and Th2 differentiation [19]. During Th1 cell differentiation, IL-2 transcription is also attenuated upon induction of T-bet. The T-bet-mediated IL-2 inhibition may affect Th cell expansion and exquisitely modulate the Th1-mediated immune response upon exposure to a pathogenic antigen.

### 3.3. Suppression of Th2 Cell Development by T-Bet

Furthermore, exogenous T-bet introduction into Th cells suppresses the production of Th2 cytokines, such as IL-4, IL-5, and IL-13, via suppression of GATA-binding protein-3 (GATA-3). Accordingly, a lack of T-bet induces spontaneous Th2 cell development in vitro and in vivo [1, 18]. The Th2-suppressive activity of T-bet was also confirmed in the absence of IFN-*γ*, indicating that T-bet has a discrete inhibitory function, independent of IFN-*γ* stimulation.

### 3.4. Other Functions of T-Bet

Recently, many studies have reported that T-bet also modulates other Th cell lineages, including Th17, Treg, and follicular Th (T_FH_) cells, in coordination with many transcription factors, such as the retinoic acid-related orphan receptor-*γ*t (ROR*γ*t), runt-related transcription factor 3 (RUNX3), and B-cell lymphoma-6 (BCL6) [20–25]. These findings suggest that T-bet is a transcription factor that is critical for fine-tuning Th cell development.

## 4. Posttranslational Modification of T-Bet

T-Bet functions as a multitasking player in the regulation of Th cell differentiation. However, the molecular mechanisms that underlie the stimulatory and inhibitory activity of T-bet in regulating target gene expression remain to be clarified. Many multitasking proteins are known to undergo posttranslational protein modifications and to determine cell fates by exerting direct stimulatory and indirect inhibitory activity on target gene expression [26, 27].

### 4.1. Tyrosine Phosphorylation of T-Bet

Antibody-based detection of T-bet proteins in western blots results in multiple bands, suggesting the posttranslational modification of T-bet in TCR-triggered Th cells. Tyrosine phosphorylation of T-bet protein occurs primarily during the early stages (days 2 to 3) of Th cell development, upon TCR engagement, and declines afterwards. Treating Th cells with the tyrosine phosphatase inhibitor pervanadate enhances the tyrosine phosphorylation of T-bet. T-Bet is mainly localized in the nucleus, and the nuclear tyrosine kinase IL2-inducible tyrosine kinase (ITK) was identified as the responsible upstream tyrosine kinase. ITK deficiency prevents tyrosine phosphorylation of T-bet in Th cells after stimulation with TCR and IL-12. Mutational research has revealed that tyrosine residue 525 (Y525) is the relevant phosphorylation site and that phosphorylation at this site plays an important role in the interaction with GATA-3. Although T-box domain in T-bet is important for DNA and protein-protein interaction, tyrosine phosphorylation of Y525 is prerequisite for the suppression of GATA-3-mediated Th2 cell differentiation. Blockade of Y525 phosphorylation abrogates the interaction with and suppression of GATA-3, resulting in impairment of Th2 suppression [5].

Furthermore, another nuclear tyrosine kinase, c-Abl, induces phosphorylation of T-bet at tyrosine residues 219, 265, and 304 in mouse T-bet [28]. A deficiency in c-Abl as well as mutation of T-bet at these residues (Y219/265/304F mutants) leads to a failure to increase IFN-*γ* induction and to suppress Th2 cytokine production, due to the loss of phosphorylation at these tyrosine residues. This, in turn, results in the aggravation of allergic lung inflammation in vivo [28]. These findings suggest that ITK- and c-Abl-induced tyrosine phosphorylation of T-bet is essential for the modulation of Th2 cell development and the allergic immune response.

### 4.2. Serine Phosphorylation of T-Bet

Although T-bet-mediated suppression of Th2 cell development is impaired by mutation of Y525 and the absence of c-Abl kinase, IL-2 suppression is retained with a Y525-mutant T-bet, suggesting the existence of an additional regulatory mechanism for IL-2 modulation [5]. Interestingly, the appearance of multiple bands of T-bet protein on western blots could be eliminated by the addition of calf intestinal phosphatase, which predominantly eliminates phosphorylation at serine/threonine residues. Mass spectrometric analysis then revealed serine 508 (S508) as another phosphorylation site in T-bet. Mutation of S508 abolishes casein kinase- (CK-) and glycogen synthase kinase-3 (GSK-3-) mediated phosphorylation in T-bet, as well as the IL-2–suppressive activity of the protein. Moreover, S508 phosphorylation is important for the interaction of T-bet with NF-*κ*B p65 and for prevention of binding of NF-*κ*B p65 to the* IL2* promoter. In accordance with the function of T-bet as an NF-*κ*B p65 inhibitor, T-bet-null Th1 cells sustain NF-*κ*B p65 activity during Th1 cell differentiation and thus produce more IL-2. Therefore, it has been suggested that T-bet is a physiological inhibitor of IL-2 during Th1 cell differentiation, through S508 phosphorylation-dependent suppression of NF-*κ*B p65.

### 4.3. Threonine Phosphorylation of T-Bet

Very recently, threonine 302 (T302) was characterized as a novel phosphorylation site in T-bet [29], although it remains unclear which kinase and phosphatase affect the phosphorylation of this residue. However, restoration of T-bet-null Th cells with a T302-mutant T-bet stimulated IFN-*γ* production as much as did wild-type T-bet; however, the mutant failed to suppress IL-2 and other Th2 cytokines. Further analysis demonstrated that T302 phosphorylation is required for the interaction of T-bet with nuclear factor of activated T cells (NFAT) and for downregulation of NFAT-mediated IL-2 and Th2 cytokines, such as IL-4, IL-5, and IL-13. NFAT is not crucial for induction of IFN-*γ* production and T302-mutant T-bet is able to bind to the* IFNG* promoter; thus, IFN-*γ* production was comparable between wild-type and mutant T-bet. In other words, mutation of T302 abrogated the T-bet-mediated suppression of IL-2 and Th2 cytokine production but did not affect the DNA-binding and IFN-*γ*-stimulatory activity of T-bet.

Indeed, T302 is located in the DNA-binding T-box, as is Y304 [28]. The T-box domain consists of several repeats of *β*-strands and *α*-helices and is involved in both dimerization and DNA binding [16]. Muller and Herrmann predicted that the *α*-helices *α*H3 and *α*H4 in brachyury (T) are important for the direct interaction of this protein with the minor and major grooves of DNA [16]. However, T302 may not be associated directly with the DNA grooves, regardless of its phosphorylation status. It would be interesting to know which upstream kinase and phosphatase regulates T302 phosphorylation and whether T302 phosphorylation affects other protein modifications of T-bet.

### 4.4. Ubiquitination of Lysine 313 in T-Bet

T-bet expression is critical for the transcriptional regulation of* IFNG* and for the development of Th1 cells, but the means of regulation of T-bet at the protein level is yet to be identified. Jang et al. have recently reported that T-bet undergoes ubiquitination-mediated proteasomal degradation during the later stages of Th1 cell differentiation [29]. Of the 16 lysine residues present in mouse T-bet protein, 11 are predominantly located within the T-box domain, and the remaining 5 are located at the carboxyl-terminus (residues 326 through 530), while no lysine residues are present in the amino-terminus (residues 1 through 134). Interestingly, lysine residues within the T-box domain are preferentially ubiquitinated upon overexpression of ubiquitin. Further analysis has identified that mutation of lysine 313 (K313) decreases ubiquitination-mediated T-bet degradation and enhances the expression level of T-bet in the nucleus and the cytoplasm. Despite the increased levels of the K313 mutant, this mutation completely abrogated T-bet functions involving DNA binding, transcriptional activation of* IFNG*, and suppression of IL-2 and Th2 cytokine production. The crystal structure of the *α*-helices of the T-box domain bound to DNA strongly suggests that the amino group of K313 is associated with the phosphate of a DNA base via hydrogen-bond interaction. In addition, mutation of K313 also leads to failure to suppress IL-2 and Th2 cytokine production; however, the interaction with and suppression of GATA-3 and NF-*κ*B p65 are not altered by mutation of K313. Interestingly, NFAT interaction is abolished in K313-mutant T-bet, which is also strongly associated with an absence of phosphorylation at T302. It is not clear yet whether and how K313 regulates T302 phosphorylation and vice versa.

## 5. T-Bet in Inflammatory and Autoimmune Diseases

Since Mosmann et al. discovered Th1 and Th2 subsets that produce signature cytokines, IFN-*γ*, and IL-4, IL-5, and IL-13, respectively, and that modulate inflammatory and allergic immune responses [30], further studies have identified novel subsets of Th cells, such as Th17, T_FH_, and Treg cells [11, 21, 23, 24, 31–34]. Extensive studies have also characterized the cytokine signaling pathways and transcription factors involved in the regulation of immune responses to pathogens [1, 35–54]. Importantly, T-bet plays a fundamental role in controlling differentiation of several subsets of Th cells and in modulating inflammatory and autoimmune diseases [10–12].

T-Bet also functions as an antiasthmatic regulator. A deficiency in T-bet spontaneously leads to the development of asthmatic symptoms, which is characterized by increased eosinophil infiltration into the airway, mucus-secreting goblet cell hyperplasia, and chronic airway remodeling with collagen accumulation and proliferative myofibroblasts; these features are often also seen in asthmatic patients [55–57]. Restoration of T-bet expression shifts the immune balance to a Th1 response and prevents and attenuates pathologic lung inflammation in vivo [58, 59].

Moreover, T-bet is protected against intracellular pathogenic infections. Abrogation of T-bet-induced IFN-*γ* production resulted in higher susceptibility to intracellular pathogens in vivo, including* Mycobacterium tuberculosis*,* Leishmania major*, and* Salmonella typhimurium* [18, 60, 61], emphasizing the importance of IFN-*γ* production by T-bet-expressing Th1 cells in the defense against bacterial infections. However, T-bet-deficient mice are resistant to infection by* Listeria monocytogenes*, because INF-*γ* production by natural killer cells is both necessary and sufficient for the host defense against* L. monocytogenes* [62].

Furthermore, T-bet can aggravate the development of inflammatory and autoimmune diseases, including inflammatory bowel disease, experimental autoimmune encephalomyelitis, inflammatory arthritis, and type I diabetes, as these inflammatory diseases are attenuated in the absence of T-bet [6, 56, 63, 64]. These findings suggest that fine-tuning of the immune response by modulation of T-bet expression could have beneficial effects for patients with chronic asthma, inflammatory bowel disease, arthritis, multiple sclerosis, and diabetes.

## 6. Conclusions and Perspectives

**Figure 2:**
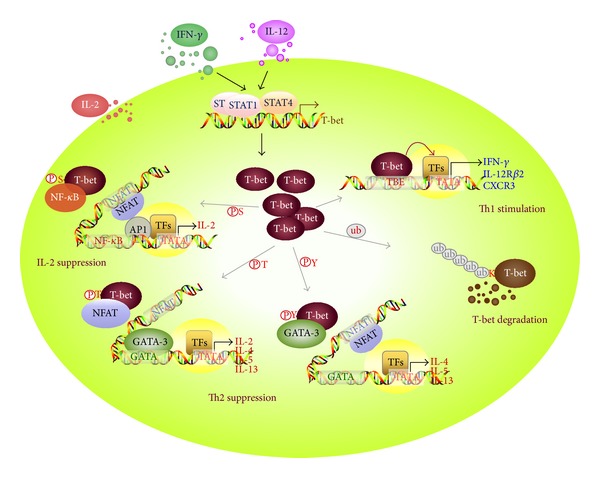
Multiple T-bet functions playing a role in Th cell differentiation. Induction of T-bet expression through activation of STAT1 and STAT4 directly stimulates the transcription of T-box-binding element-containing genes, such as* IFNG*,* IL12RB2*, and* CXCR3*, thereby enhancing Th1 cell development. T-Bet undergoes serine phosphorylation at S508 and then downregulates IL-2 production in Th1 cells by recruiting NF-*κ*B p65 from the* IL2* promoter. Protein levels of T-bet in Th1 cells can be controlled by the ubiquitin-mediated proteasomal degradation pathway. Moreover, T-bet protein undergoes additional posttranslational modifications, for example, phosphorylation at T302 and Y525, which facilitates its suppression of the Th2 cytokine production that is induced by activation of NFAT and GATA-3.

T-bet is a T-box domain-containing transcription factor that is typically involved in developmental regulation but exerts multiple functions in Th cell differentiation; transcriptional activation of IFN-*γ*-expressing Th1 cells, indirect suppression of Th2, Th17, and Treg cell development, and fine-modulation of IL-2 production in Th1 cells. This multitasking is not surprising, as T-bet undergoes several posttranslational modifications. Phosphorylation at Y525 plays a role in GATA-3 suppression during Th2 regulation, phosphorylation at S508 causes NF-*κ*B p65 suppression in the context of IL-2 regulation in Th1 cells, phosphorylation at T302 plays a role in fine-tuning IL-2 production, and ubiquitination at K313 plays a role in controlling T-bet protein stability. Thus, posttranslational modification of T-bet facilitates its functional diversity and the complexity of its modulation of cytokine expression (Figure 2). It is not known whether the various posttranslational modifications occur sequentially or simultaneously, whether one type of protein modification affects other modification, or whether changes in the posttranslational modification of T-bet are related to the development of infectious and chronic inflammatory diseases. Further identification of novel protein modifications related to T-bet functions would provide valuable insights into the development of powerful therapeutic interventions for controlling chronic inflammatory and autoimmune diseases.

## List of Abbreviations

BCL-6: B-cell lymphoma-6
CK: Casein kinase
GATA-3: GATA-binding protein 3
GSK-3: Glycogen synthase kinase-3
IFN: Interferon
IL: Interleukin
ITK: IL-2-inducible T cell kinase
NFAT: Nuclear factor of activated T cells
NF-*κ*B: Nuclear factor kappa B
ROR*γ*t: Retinoic acid-related orphan receptor gamma t
RUNX: Runt-related transcription factor
STAT: Signal transducer and activator of transcription
T-bet: T-Box protein expressed in T cells
Th: T helper
Treg: Regulatory T
T_FH_: Follicular T helper
Ub: Ubiquitin.
